# Genetic diversity and population structure in Ethiopian *Urochloa brizantha* genotypes uncovered using inter simple sequence repeat (ISSR) markers

**DOI:** 10.1371/journal.pone.0340368

**Published:** 2026-01-08

**Authors:** Fentahun Meheret, Kassahun Tesfaye, Teklehaimanot Haileselassie, Meki Muktar, Dereje Worku, Bimrew Asmare, Mengistie Taye

**Affiliations:** 1 College of Agriculture and Environmental Sciences, Bahir Dar University, Bahir Dar, Ethiopia; 2 Institute of Biotechnology, Bahir Dar University, Bahir Dar, Ethiopia; 3 Institute of Biotechnology, Addis Ababa University, Addis Ababa, Ethiopia; 4 Feed and Forage Development, International Livestock Research Institute, Addis Ababa, Ethiopia; National Cheng Kung University, TAIWAN

## Abstract

*Urochloa brizantha* is a tropical C4 grass belonging to the *Urochloa* genus. Despite its immense potential as animal feed and its contribution to livestock production in sub-Saharan African countries, particularly Ethiopia, its utilization remains limited due to an inadequate supply of *Urochloa* cultivars and an insufficient genetic characterization of currently available genotypes. Hence, this study was conducted to assess the genetic diversity and population structure of 66 *U. brizantha* genotypes from the Ethiopian collection by leveraging six polymorphic inter-simple sequence repeat (ISSR) markers. These markers generated a total of 80 scoring bands, with 79 of which were polymorphic, and an average of 13.33 bands per primer. The polymorphism information content (PIC) ranged from 0.31 to 0.34, revealing the significance of these ISSR markers in uncovering highly polymorphic loci across the genotypes. Analysis of Molecular Variance (AMOVA) revealed that 69.68% of the genetic variability was distributed within the population, while the remaining variation was among populations. Genotypes collected from Illubabor revealed the highest degree of genetic diversity (PPL = 81.25% and Shannon’s information index = 0.34 ± 0.24), while the genotypes from central Gondar exhibited the least genetic diversity (PPL = 51.25% and Shannon’s index = 0.28 ± 0.27). The 66 genotypes of *U. brizantha* were grouped into seven clusters (K = 7) based on STRUCTURE analysis and the unweighted pair group method with arithmetic mean (UPGMA) algorithm after the formation of three major clusters at the root. These genotype collections generally formed their own cluster following their regional collection, though cluster analysis also revealed genetic admixture. These results illustrated the extent of genetic diversity within Ethiopian *U. brizantha* genotypes, confirming their potential for conservation and forage improvement. This could provide an alternative source of animal feed in sub-Saharan Africa, where feed shortages are a significant constraint to sustainable livestock production.

## Introduction

Ethiopia has the largest livestock population in Africa, supporting the livelihoods of over 11.3 million rural households [1]. However, livestock productivity is vulnerable due to inadequate feed supply in terms of both quantity and quality [2]. Approximately 94% of the country’s annual dry matter feed supply comes from natural pasture (56.83%) and crop residues (37.37%) [3]. Although these are commonly used feed resources, they have been classified as poor quality [1,2]. Despite these being a common feed resource for livestock, the FOA reports (2018) also indicate shortages of feed resources on a dry matter basis, with 51.7% of the metabolizable energy and 48.2% of the crude protein [4]. Therefore, incorporating improved forage species, which currently have a utilization rate of not more than 2%, into the feed supply is a vital approach to alleviating feed shortages to promoting sustainable livestock production [1,3,5].

*Urochloa* grasses (also known as *Brachiaria* grasses) are one of the alternative forage species, having been identified as an important, high-yielding and environmentally friendly genus and gaining considerable recognition as a climate-smart option for integrated tropical agriculture, including in Ethiopia [6–8]. *Urochloa brizantha (A. Rich.) Stapf (syn. Brachiaria brizantha)* is one of the *Urochloa* grasses, which belong to the *Urochloa* genus, which encompasses about 100 species [9]. This species is distributed naturally across African savanna grasslands, woodland edges, and dense thicket ecosystems within Sub-Saharan African countries [9]. Among the *Urochloa* grasses*, U. brizantha* is recognized as one of the commercially most promising cultivar sources grown in tropical regions, thus supporting millions of livestock [10]. In SSA, particularly in Ethiopia, *Urochloa brizantha* occurs naturally between 1,100 and 2,100 meters above sea level in the former regions of Tigray, Shewa, Gojjam, Gondar, Kefa, Gamo Gofa, Sidamo, Bale, Illubabor and Harerge [11]. However, it remains an underutilized source of livestock feed in Sub-Saharan African countries [5,12].

*U. brizantha* is a perennial C4 grass, reproduces mainly through apomixis in its polyploid forms (2n = 4x, 5x, or 6x) and sexually in its diploid form (2n = 2x) [13,14]. The species is known for its various significant traits, including resilience to stress conditions of pests, diseases, infertility soil, acidic soil, drought and waterlogging [15,16]. It also provides high dry matter yield at low precipitation and is nutritious to livestock within SSA countries [17,18]. Hence, expanding the cultivation of *U. brizantha* cultivars in Ethiopia could alleviate the country’s shortage of livestock feed, particularly the current deficits of 9% in dry matter and 42% in crude protein [4]. However, the supply of *Urochloa* cultivars adapted to the specific pest and disease challenges in Sub-Saharan African countries is inadequate [19,20].

Genotypes of *Urochloa* grasses collections were made extensively between 1985 and 1986 with the support of international institutions within various regions of SSA [21]. Of which the International Livestock Research Institute (ILRI) gene bank has had a lion’s share in maintaining the genotype/accession collections for decades. These collections of genotypes are potential genetic resources for developing specific cultivars that can be adapted to regions of Sub-Saharan Africa (SSA) through the employment of effective breeding programs [22]. Studying the genetic diversity and population structure of these *Urochloa* grasses using molecular marker techniques would help the efficiency and effectiveness of grass breeding programs [22–25]. Of which ISSR markers assessed the molecular variability of 93 *U. ruziziensis* genotypes from the EMBRAPA collection, revealing significant genetic diversity and similarity coefficients ranging from 0.0 to 0.95 [22]. The SSR markers were also employed to analyze genetic diversity and population structure in *U. humidicola*, revealing that its germplasm collection formed four distinct main clusters [26]. Additionally, RAPD markers revealed clear genetic dissimilarities among *Urochloa* species accessions, highlighting the variations between different *Urochloa* genotypes [27]. Similarly, SSR markers have been used for the genetic characterization of *U. brizantha* genotypes, with studies generally revealing three major clusters and gene pools in germplasm collections [28,29]. However, the levels of genetic diversity detected using SSR marker varied, where it was reported as high for genotype collection from Ethiopian [29] and Kenyan [30] studies, and there was no substantial difference among the genotype collections at the pools maintained at EMBRAPA Beef Cattle, despite originating in Sub-Saharan Africa (SSA) [28]. Besides this, the SSR marker system, while effective, involves the complex generation of simple sequence repeat (SSR) markers based on pre-sequencing information, which makes the development of SSR markers lengthy and potentially costly when compared to other markers [31,32].

In developing countries in SSA with limited research resources, such cots limit the use of SSR to study initial collections of genetic resources. Therefore, incorporating alternative molecular markers, such as inter simple sequence repeat markers, may overcome these limitations and improve our understanding of the species’ genetic background. The primer motifs used in ISSR are composed of di-, tri-, tetra-, and penta-nucleotide sequences and sometimes include anchored degenerate bases in the adjacent DNA regions [33]. Researchers use these markers to identify unique parts of genomic DNA found between two microsatellite repeats with opposite configurations [34]. This would enhance the management of its genetic resources for breeding programs and conservation initiatives in the region of SSA. Despite the limitations of ISSR markers, such as non-specificity bindings and unable to detect heterozygous in comparison to the SSR marker system, ISSR markers were substantially used in the detection of high genetic variability during genetic assessments of Napier grass and Pearl millet [35], Bermuda grass [36], Ryegrass [37] and *U. ruziziensis* [22]. These techniques are generally used in many significant areas of biological and agricultural studies, including genetic diversity, phylogeny, evolution, and genomic mapping [38–40]. This study was consequently conducted to assess the genetic diversity and population structure of *U. brizantha* genotypes collected from Ethiopia, using inter-simple sequence repeat (ISSR) markers.

## Materials and methods

### Plant materials

In this experiment, a total of sixty-six *Urochloa brizantha* genotype samples were used in this experiment (Table 1). The samples were collected from various regions of Ethiopia and grouped into seven populations and maintained at ILRI. The samples were obtained from the field gene bank of ILRI, Ziway conservation sites; a site located 1,640 meters above sea level, situated 165 kilometers south of Ethiopia’s capital city, Addis Ababa (7° 53’ 9“ N, 38° 44’ 68” E). Ziway has an average yearly temperature ranging from 20°C to 26°C and receives 700 mm of rainfall per year. The predominant soil type is sandy loam, with a pH level of 8.04.

**Table 1:** Number of samples and their origin by collection region across different parts of Ethiopia.

Populations*	Sample size	Region	Origin of collection
**Wollega (west and east)**	11	Oromiya	Western Ethiopia
**Jimma**	11	Oromiya	Western Ethiopia
**Borena**	8	Oromiya	South Ethiopia
**Illubabor (Ilu Ababora)**	11	Oromiya	Western Ethiopia
**North Omo**	9	South West Ethiopia	South Ethiopia
**West Gojjam**	8	Amhara	Northwestern Ethiopia
**Central Gondar**	8	Amhara	Northwestern Ethiopia
**Total**	66	7	

*Population clustering/grouping is made based on the potential of genotype collections, purposely to see the genetic structure.

### Genomic DNA extraction and PCR amplification

A total of three young leaves of genotypes (S3 File) were harvested and dried using blue silca gel. Genomic DNA was extracted from these leaves using a modified CTAB procedure [41]. The concentration and purity of genomic DNA (1.8μ/ml) were measured using a NanoDrop device. A 26 μl reaction volume was used for DNA amplification in a Biometra 2003 T3 Thermocycler, following screening of six out of 13 ISSR primers (Table 2), which were previously used in the study of the genus *Urochloa* [22] and other grasses [36,42]. A 1.67% agarose gel was used to separate the PCR products [43]. Then, it was stained with ethidium bromide solution for 10 minutes and destained with deionized H₂O for 30 minutes (with slight shaking) to allow for clear visualization of bands. The image was captured using a high-resolution camera equipped with a gel documentation system, and it was documented for future use.

**Table 2:** List of primers and their diversity parameters used in this study.

ISSR-Primer	Primer motif	T (°C)	Amplification quality	Repeat motif	TSB	PIC
**UBC-818**	(CA)8G	48	Polymorphic, reproducible	Dinucleotide	15	0.32
**UBC-844**	(CT)8RC	48	Polymorphic, reproducible	Dinucleotide	12	0.33
**UBC-841**	(GA)8YC	48	Polymorphic, reproducible	Dinucleotide	16	0.34
**UBC-812**	(GA)8A	45	Polymorphic, reproducible	Dinucleotide	19	0.31
**UBC- 880**	(GGAGA)3	45	Polymorphic, reproducible	Penta-nucleotide	10	0.34
**UBC- 873**	(GACA)4	48	Polymorphic, reproducible	Tetra-nucleotide	8	0.31

Single-letter abbreviations for mixed base positions: R = (A, G) Y = (C, T); Source: Primer kit 900 (UBC 900), University of British Colombia, Adenine (**A**), Guanine (G), Cytosine(C) and Thymine(T); total sorbale bands (TSB); polymorphism information contents (PIC) and annealing temperature(T (°C).

### Data collection and analysis

The data matrix (S1 File) was generated by assigning a score of a present (1), absent (0), and missing (?) to each lane loaded with PCR product of genotype (S4 File) based on the standard band scoring procedure [44,45]. The PIC, observed number of alleles (Na), and number of effective alleles (Ne) of the primers were computed using PowerMarker version 3.25 with these data [46]. The PopGen version 1.31 software applications [47] were also used to calculate the percent polymorphic loci (PPL), gene diversity (h), and Shannon’s information index (I), as well as the genetic differentiation of populations. The population structure was constructed using the STRUCTURE 2.3.4 program under the assumption of no admixture [48]. These structure analysis settings were adjusted for each ‘K’ value run (from 1 to 10), with 100,000 replicates simulated for the burn-in period and the Markov chain Monte Carlo (MCMC) processes. A total of 10 iterations were used for each K-value due to the undefined number of populations. Then, the analytical products were harvested using Harvester-Selector, web-based software [49]. The peak K was selected as the optimal number of clusters based on the probability of the data, Ln P (K), which was plotted against K-values. Moreover, the genetic variance distribution in the population was estimated using 1,000 permutations with Arlequin software, version 3.1 [50]. The Nei’s 1983 correlation coefficient of genotypes was calculated using PowerMarker software, version 3.25 [46]. The relationship of genotypes was constructed using the unweighted paired group method with arithmetic average (UPGMA) method with this software application. The resulting UPGMA tree was displayed and edited using MEGA version 11 [51].

## Results and discussion

### Polymorphism of the ISSR markers

In the present study, six primers were eligible for the final amplification of 66 genotypes of sampled DNA due to their reproducibility and rate of polymorphism during the screening and optimization stage. These primers produced a total of eighty (80) scorable bands, and 79 of which were polymorphic (Table 3). In this study, primer UBC-812 produced the maximum number of total scorable bands (19 TSB), while primer UBC-873 produced the minimum (8 TSB), as illustrated in Table 2. The primers used in this study were able to generate an average of 13.33 bands per primer, which is greater than in a previous study of *Cynodon dactylon* (local turf grass), which used 28 ISSR primers to generate an average of 9.17 bands per primer [52]. The number of scorable bands generated by the six primers is nearly equal to the number of scorable bands (89) reported when the ISSR marker was used to amplify DNA from *U. ruziziensis* genotypes [22]. However, the primers used in the current study generated a maximum of 80 scorable bands, which is higher than the 68 TSB produced from *Urochloa brizantha* cv [53]. The number of bands scored in the current study is also comparable to the number of bands scored using ISSR markers in cool-season grass species [23] and in *Lolium persicum* grasses, as reported in previous studies [54].

**Table 3:** Diversity levels of genotypes within seven populations.

Population	Sample Size	NPL	PPL%	Na ± SD	Ne ± SD	h ± SD	I ± SD
**Wollega**	11	54	67.5	1.68 ± 0.47	1.34 ± 0.33	0.21 ± 0.18	0.33 ± 0.26
**Jimma**	11	46	57.50	1.58 ± 0.50	1.34 ± 0.39	0.20 ± 0.20	0.30 ± 0.29
**Borena**	8	53	66.25	1.67 ± 0.48	1.42 ± 0.36	0.25 ± 0.20	0.37 ± 0.28
**Illubabor**	11	65	81.25	1.81 ± 0.39	1.39 ± 0.34	0.24 ± 0.18	0.34 ± 0.24
**North Omo**	9	48	60	1.60 ± 0.49	1.35 ± 0.35	0.21 ± 0.19	0.31 ± 0.28
**West Gojjam**	8	48	60	1.60 ± 0.49	1.35 ± 0.36	0.21 ± 0.20	0.31 ± 0.28
**Central Gondar**	8	41	51.25	1.56 ± 0.50	1.31 ± 0.34	0.19 ± 0.19	0.28 ± 0.27
**Overall**	66	79	98.75	2.0 ± 0.11	1.67 ± 0.29	0.38 ± 0.12	0.56 ± 0.15

SD = standard deviation; Number of polymorphic loci (NPL); Percent polymorphic loci (PPL%); observed number of alleles(Na); number of effective alleles(Ne;, Nei’s (1973) gene diversity (h) and Shannon‘s information Index(I)

In the current ISSR marker analysis, the UBC-873 and UBC-812 primers had a PIC value of 0.31. On the contrary, UBC-841 and UBC-880 had high PIC values calculated at 0.34. The selected primers used to amplify the DNA of genotypes from *U. brizantha* showed PIC values ranging from 0.31 to 0.34, which is within the range of 0.24 to 0.48 PIC values of ISSR reported from plant species [55–57]. The current study aligns with the previous one that employed ISSR markers, demonstrating comparable levels of polymorphism with PIC values ranging from 0.29 to 0.35 during the screening of twenty-four mulberry accessions [58]. However, the PIC values in the current study differ from those reported for other marker systems in wheat grass species, which range from 0.44 to 0.81 [38,59]. These values are also comparable to the 0.34 PIC values observed in the genome of *Setaria* species employing 8 ISSR markers [60]. Hence, the primers selected for the current study had moderate PIC values for UBC-873, UBC-812 and UBC-818, and high values for UBC-841, UBC-880, and UBC-844, based on the proposed range of PIC values for plant species, which are considered to be informative [55,56]. These results confirmed that the ISSR marker evaluation in the current study revealed different polymorphism rates for the primers, likely due to the high genetic diversity of the Ethiopian genotypes. This finding is consistent with previous analyses of Ethiopian genotypes, which revealed high genetic diversity using 23 SSR markers [29].

### Genetic diversity among genotypes

In the current study, a total of 66 genotype collections from various parts of Ethiopia were assessed for their genetic profile using six ISSR markers, as presented in Table 3. The Illubabur genotypes displayed the highest degree of PPL (81.25%) and Shannon’s information index (0.34 ± 0.24) than genotypes within the proposed seven populations. However, this genotype had an effective allele frequency of 1.39 ± 0.34 and a gene diversity of 0.24 ± 0.18 (Nei 1973), placing it second in diversity among those assessed genotypes. Wollega genotypes also had a PPL of 67.5% and Shannon’s information of 0.33 ± 0.26, which indicated that Wellega has the second most diversified genotype set. However, the Central Gondar genotypes unveiled low genetic diversity (PPL = 51.25, Ne = 1.31 ± 0.34, I = 0.28 ± 0.27, and h = 0.19 ± 0.19). In this study, the diversity index results showed that ISSR markers in the genomic DNA of the Ilubabor genotypes detected a high degree of genetic diversity, but the genotypes from the Central Gondar had the least. These genetic profile variations among genotypes likely stem from the differential amplification of multiple ISSR marker sites (e.g., presence/absence or band patterns) across the Illubabor genotypes’ genomes, contrasted with the more restricted primer binding in Central Gondar accessions. The abundance of such ISSR sites in a species’ genome often signals underlying genetic rearrangements, such as structural polymorphisms or microsatellite instability [61,62]. Besides this, it might be related to genetic material flow due to migratory agents, and limitation of genotype sharing due to barriers such as forest areas, mountains, and apomictic reproduction, which might explain the high genetic diversity observed in the Illubabur genotypes collection. On the other hand, the ISSR region of the site is conserved in the genome of the species due to asexual reproduction, cultivar development, and limited species distribution [53,63,64]. This low level diversity might be due to genotype-narrow collection coverage, frequent inbreeding and gene flow from markets, limited barriers and poor adaptability. North Omo and West Gojjam genotypes, on the other hand, exhibited similar polymorphism loci (60%), gene diversity (0.21), and Shannon’s information index (0.31) values; even though they did not have similar cluster groups from cluster analysis. The two genotypes are geographically isolated. The two genotypes are geographically isolated; the similarity may be due to identical genomic regions amplified and the size of bands separated by gel electrophoresis.

In this study, the total genetic diversity (Ht) and genetic diversity within g populations (Hs) were estimated at 0.37 ± 0.01 and 0.21 ± 0.01, respectively. This amount of the population’s genetic diversity, as measured by ISSR marker analysis, indicates diversification of genotypes to be considered during conservation and breeding initiatives. This finding is consistent with those reported when 23 SSR markers were used for 112 Ethiopian accessions [29] and 79 Kenyan ecotypes were assessed using 22SSR markers [30].

The analysis of molecular variance (AMOVA) for the seven populations proposed for this study detected significant genetic variation (p < 0.001) among populations, as shown in Table 4. Of the total variation component, high values were recorded within populations, accounting for 69.68% of the total genetic variation caused by differences in genotypes within populations. The remaining 30.32% was distributed between genotypes (Table 4) within populations. These results propose that genetic differences in genotypes may be due to differences in the samples’ origin and how genotypes reproduce across Ethiopia’s different ecological regions. The results of this study are consistent with previous investigations of molecular variation within *Urochloa* genotypes in Uganda [65], *U. brizantha* accessions and six cultivars [31], *U. ruziziensis* genotypes [22] and *Lolium persicum* species [54]. Similar patterns were also observed in non-grass species such as Arabica coffee (*Coffea arabica L*.) [66]. Furthermore, the fixation index (FST) value of 0.30 found in this study is higher than the previously reported value of 0.15, indicating significant population differentiation [67]. This differentiation likely results from genotypic adaptation and reproductive isolation across regions, mainly driven by the geographical distance between western and southern Ethiopia. These aspects are significant drivers of the genetic differences observed among groups, as supported by studies on regional genotype-environment interactions [68, 69].

**Table 4:** Analysis of Molecular Variance (AMOVA) among and within seven populations of *U. brizantha* genotypes.

Source of Variance	df	Sum of Squares	Variation components	Percentage of variation	p-value
**Among Population**	6	190.288	2.71252 Va	30.32	0.00000
**Within Population**	59	367.773	6.23344 Vb	69.68	
**Total**	65	558.061	8.94595		
**Fixation Index**	FST: 0.30321				

### Population structure based on admixture analysis

**Fig 1:**
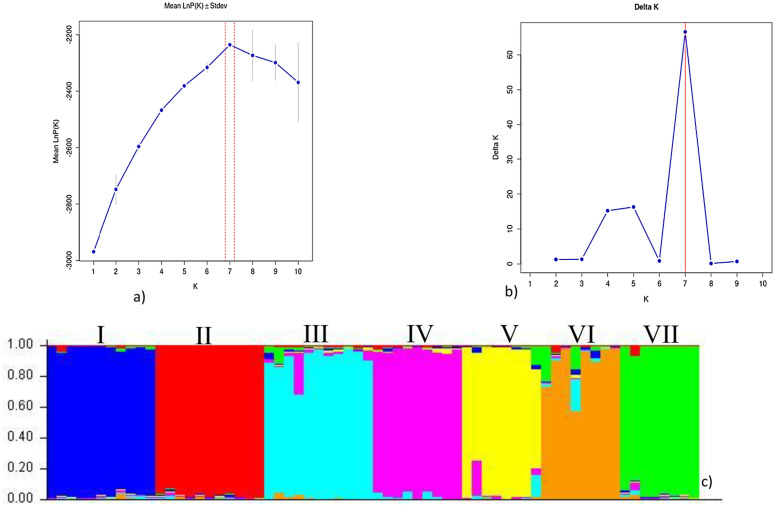
Population structure analysis of U. brizantha genotypes. **(a)** Log probability of the data, Ln P(K) (± SD), plotted against the number of clusters (K); **(b)** ΔK values derived from mean log probabilities across STRUCTURE runs for K = 1–10; **(c)** Clustering at the optimal K = 7, where most genotypes grouped by collection region. Membership coefficients are shown on the left y-axis (vertical bar); multi-colored segments indicate admixture proportions within genomes. Lower x-axis labels denote the 66 U. brizantha genotype samples. The seven inferred populations are: Wellega (I), Jimma (II), Borena (III), Illubabor (IV), North Omo (V), West Gojjam (VI), and Gondar (VII).

**Fig 2:**
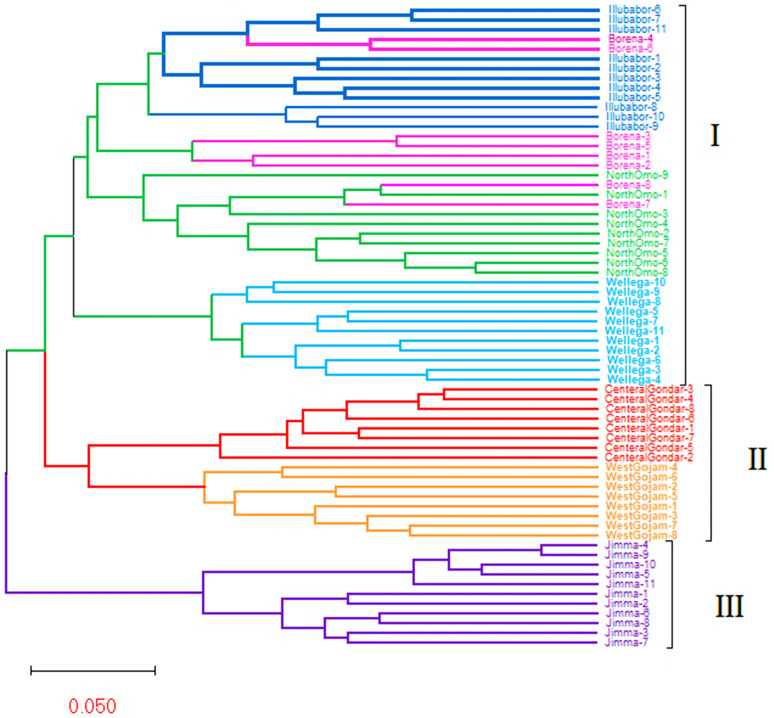
UPGMA-based dendrogram visualization for sixty-six genotypes clustered into three major clusters (I, II and III). This dendrogram showed that Cluster-III only contained genotypes of Jimma, cluster-II divided into two sub- clusters of genotypes of central Gondar and west Gojjam whereas cluster-I contained four sub-clusters of genotypes Wellega, north Omon, Borena and Illubabor where the seven sub-clusters (highlighted with seven colours) of *U. brizantha* derived from diverse ecosystems of Ethiopia utilizing the amplification result of six ISSR primers.

A total of sixty-six genotype collections were regrouped into seven separate clusters of branches at the peak K value (K = 7). These clusters mainly referred to genotypes in their collection regions at the sub-cluster level (shown in different colors in Fig 1). In this study, the “K” value of 7 shows a higher peak compared to the other “K” values (from 1 to 10). These results were aligned with the molecular clustering analysis using the UPGMA method, showing the following three main clusters formation at the basis and then formation of seven separate clusters (shown with seven colours in the UPGMA, Fig 2). Even though a clear genetic differentiation pattern was observed among most genotypes, the North Omo, West Gojjam, and Illubabor genotypes exhibited greater genetic admixture, as shown by the color bands in Fig 1. These admixtures mainly originated from the Borena, Illubabur, and Central Gondar genotypes. Therefore, the current analysis demonstrates that ISSR markers effectively amplify genomic DNA to classify genotypes within related genomes. This finding aligns with genomic DNA amplification studies in *Urochloa* species (*U. ruziziensis* and *U. brizantha*) using SSR markers, which revealed three major allelic pools (I, II, and III). It also produced similar results to those obtained with SSR markers in detecting admixture within the population structure [29, 31]. These genetic differences may be attributed to aspects such as chromosome number, flanking regions of amplification, reproductive modes, and ecological adaptation. The observed admixture could result from agents like animal migration or seed donations linked to Ethiopia’s culture of charity mobilization during droughts and other social disasters.

### Population structure based on UPGMA analysis

The genetic relationship between genotypes was explored using the UPGMA dendrogram algorithm grouping approach (Fig 2). It is constructed using the correlation coefficient of Nei’s (1983) method (S2 File), which grouped 66 genotypes into three major clusters on the genetic basis and branched into seven sub-clusters (Fig 2). This resembles the UPGMA pattern observed in the clustering of resistant versus susceptible wheat cultivars for common root rot disease [40]) and in the clustering of 61 N. nouchali accessions from various districts in Kerala, India, using 21 scorable inter-simple sequence repeat (ISSR) markers [57]. Furthermore, the genotypes Jimma and Illubabor showed two and three sub-cluster branch forms, respectively. The remaining five sub-cluster branches showed irregular branching patterns. In this study, genomic DNA amplification products distinguished genotypes based on the origin of samples, indicating genetic heterogeneity within selected genotypes. These results were similar to those genotypes of six grass species (cool season grass) grouped into their origin of species [23] and the separation of out-group genotypes from *U. ruziziensis* genotypes [22] using inter simple sequence repeat markers. However, the current results disagreed with *U. brizantha* accessions, which were clustered into three gene pools with no clear branches and six clusters at principal coordinates and neighbor joining (NJ) algorithm analysis, respectively, using the product of simple sequence repeat markers (23SSR) [29]. These discrepancies in results could be attributed to amplification regions and molecular marker detection capacity. Thus, the current findings indicated that the selected inter simple sequence repeat markers were operative for the classification of genotypes. Furthermore, the similar molecular genetic profiles of ecotypes, as indicated in the four Borena ecotypes, were regrouped into the clusters of Illubabor (Borena 4 and 6) and North Omo (Borena 7 and 8). These findings could be attributed to a similar region of genomic DNA amplifications, identical size PCR products or an indication of gene flow.

## Conclusion

The current study, which analyzed ISSR markers, revealed that the genotypes of *U. brizantha* obtained from different geographical regions within Ethiopia exhibited varying degrees of genetic diversity. The genotypes collected from Illubabor exhibited the highest level of genetic diversity, while those collected from Central Gondar exhibited the least. Genotypes collected from various regions of Ethiopia were organized into seven sub-clusters, each corresponding to a collection region. However, population structure analyses detected genetic admixture among the genotypes, particularly in North Omo, West Gojjam, and Illubabor. Furthermore, four Borena genotypes were found grouped into clusters belonging to Illubabor and North Omo during UPGMA analysis, rather than clustering solely with the Borena group. Consequently, the study concluded that these *U. brizantha* genotypes possess more diverse ISSR regions within their genomic structure. This confirms that *U. brizantha* genotypes are a diverse genetic resource that could be used in breeding programs to develop cultivars. Further genetic characterization of the whole genome using modern techniques is required to assess the functional genome before initiating genetic resource conservation and breeding programs in sub-Saharan Africa.

## Supporting information

S1 File Data matrix used for genetic diversity and population structure analysis. (XLSX)

S2 File Correlation coefficient of Nei’s 1983 computed from the data matrix used to construct the dendrogram UPGMA. (XLSX)

S3 File Field trial established with *Urochloa brizantha* accessions at campus of Peda, Bahir Dar University, Bahir Dar, Ethiopia (Photo credit: Fentahun Meheret, 2024). (PDF)

S4 File PCR product of ISSR markers generated from seven populations of *U. brizantha* using six selected primers. (PDF)
