# Supplementary figures and images for: Genetic diversity and population structure in Ethiopian Urochloa brizantha genotypes uncovered using inter simple sequence repeat (ISSR) markers
Source: PLoS One. 2026 Jan 8;21(1):e0340368. doi: 10.1371/journal.pone.0340368 (PMC12782424; doi:10.1371/journal.pone.0340368)

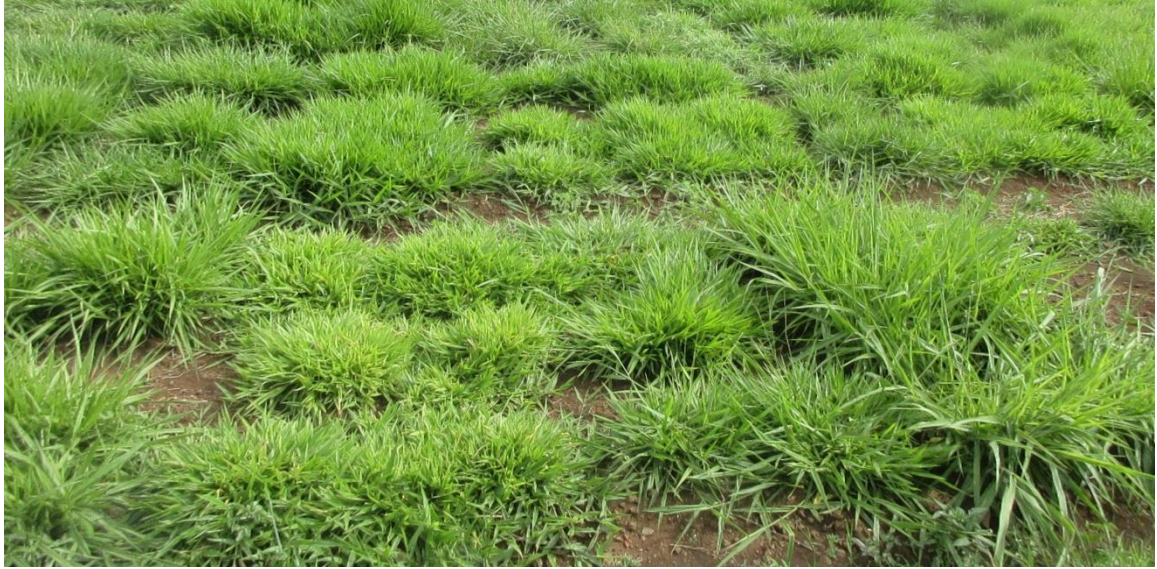

Supplement: S3 File — (PDF) [file pone.0340368.s003.pdf]

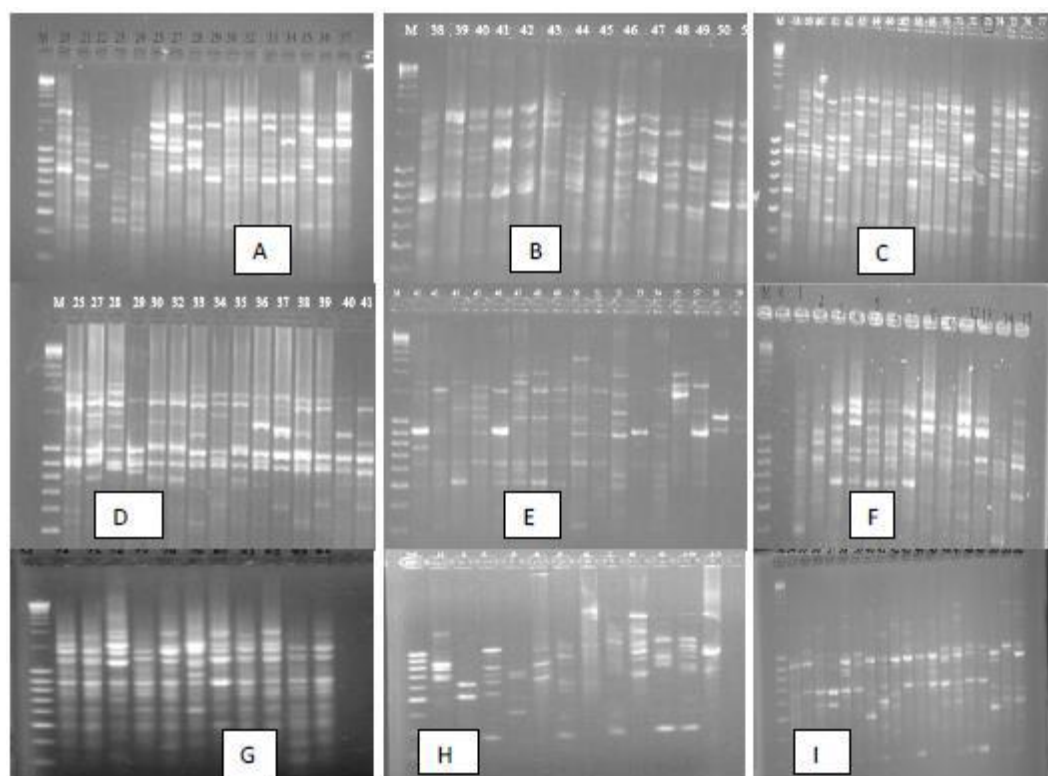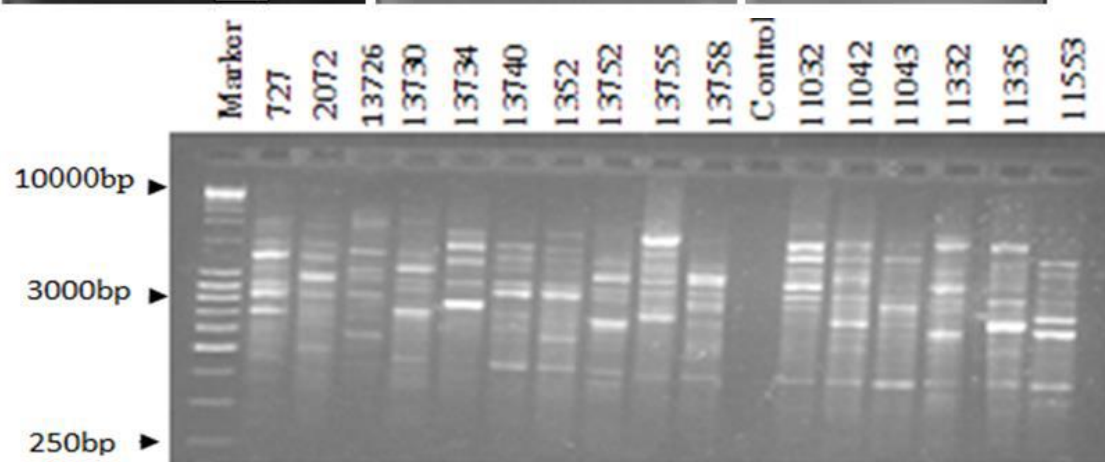

Supplement: S4 File — (PDF) [file pone.0340368.s004.pdf]
